# Fagaceae pollen from the early Cenozoic of West Greenland: revisiting Engler’s and Chaney’s Arcto-Tertiary hypotheses

**DOI:** 10.1007/s00606-014-1118-5

**Published:** 2014-08-02

**Authors:** Friðgeir Grímsson, Reinhard Zetter, Guido W. Grimm, Gunver Krarup Pedersen, Asger Ken Pedersen, Thomas Denk

**Affiliations:** 1 Department of Palaeontology, University of Vienna, Vienna, Austria; 2 Department of Palaeobiology, Swedish Museum of Natural History, Stockholm, Sweden; 3 Department of Stratigraphy, Geological Survey of Denmark and Greenland, Copenhagen, Denmark; 4 Geological Museum, Natural History Museum of Denmark, Copenhagen, Denmark

**Keywords:** Arctic, Arcto-Tertiary element, Castaneoideae, Eocene, *Eotrigonobalanus*, *Fagus*, Palynology, *Quercus*

## Abstract

In this paper we document Fagaceae pollen from the Eocene of western Greenland. The pollen record suggests a remarkable diversity of the family in the early Cenozoic of Greenland. Extinct Fagaceae pollen types include *Eotrigonobalanus*, which extends at least back to the Paleocene, and two ancestral pollen types with affinities to the Eurasian *Quercus* Group Ilex and the western North American *Quercus* Group Protobalanus. In addition, modern lineages of Fagaceae are unambiguously represented by pollen of *Fagus, Quercus* Group Lobatae/Quercus, and three Castaneoideae pollen types. These findings corroborate earlier findings from Axel Heiberg Island that Fagaceae were a dominant element at high latitudes during the early Cenozoic. Comparison with coeval or older mid-latitude records of modern lineages of Fagaceae shows that modern lineages found in western Greenland and Axel Heiberg likely originated at lower latitudes. Further examples comprise (possibly) *Acer*, *Aesculus*, *Alnus*, *Ulmus*, and others. Thus, before fossils belonging to modern northern temperate lineages will have been recovered from older (early Eocene, Paleocene) strata from high latitudes, Engler’s hypothesis of an Arctic origin of the modern temperate woody flora of Eurasia, termed ‘Arcto-Tertiary Element’, and later modification by R. W. Chaney and H. D. Mai (‘Arcto-Tertiary Geoflora’) needs to be modified.

## Introduction

Engler (1882) noted a close relationship between Cenozoic plant assemblages of the Arctic region and the modern northern temperate woody flora. Based on this he coined the term ‘Arcto-Tertiary Element’ for plant groups that today dominate in temperate forest regions of North America, Europe and East Asia. The southern counterpart of the Arcto-Tertiary Element was the so-called ‘Palaeotropical Element’ comprising lineages today found in the Old World Tropics. Based on Engler’s classification, many palaeobotanists characterised Cenozoic floras by the proportion of the Arcto-Tertiary and Palaeotropical Element in fossil plants assemblages (reviewed in Mai 1995). Chaney (1959) extended this concept into a broader context, establishing the term ‘Arcto-Tertiary-Geoflora’. Based on his observation that Cenozoic floras of the northern Pacific Basin were markedly similar, Chaney concluded that they “must all have had a common area of origin during Cretaceous and early Tertiary time at high northern latitudes, where we find their earliest record of their occurrence” (Chaney 1959). He defined a ‘geoflora’ as being “a group of plants which has maintained itself with only minor changes in composition for several epochs or periods of earth history”. Chaney stated further that these geofloras would have undergone considerable range shifts in response to changing environmental conditions and climates. This view was strongly criticised in various papers by Wolfe (1969, 1972). In contrast, Mai (1991) confirmed the geofloral concept of Chaney.

Because the (deciduous) Fagaceae comprise the dominant tree species of the northern temperate regions across the Northern Hemisphere, and have an exceptionally rich fossil record, they provide an ideal group to assess the origin of the Arcto-Tertiary Element. The original Arcto-Tertiary Element of Engler was based on the Palaeogene Arctic floras described by Heer (1868–1883). These floras were originally believed to be of Miocene age but later their Palaeogene age was established by evidence from various sources. Heer described numerous modern taxa such as *Acer* (Sapindaceae)*, Betula*, *Carpinus*, *Corylus* (Betulaceae), *Castanea, Fagus,* and *Quercus* (Fagaceae) among others, which today are typically found in the temperate mid-latitudes (Cfa to Dfb climates according to Köppen-Geiger; see Kottek et al. 2006 for all abbreviated climate types mentioned in the text). However, the generic determinations by Heer were subsequently called into question by other authors and re-assigned to extinct genera (e.g. Boulter and Kvaček 1989; Kvaček et al. 1994; Manchester 1999). At the same time, a substantial number of modern woody angiosperm genera have a reliable fossil record from Arctic regions (*Acer, Aesculus* [Sapindaceae], Manchester 2001; Budantsev and Golovneva 2009; *Fagus, Quercus,* McIntyre 1991; McIver and Basinger 1999; Denk and Grimm 2009; *Ulmus* [Ulmaceae], Denk and Dillhoff 2005; Budantsev and Golovneva 2009; Wang et al. 2010).

In this paper we present a new and rich palynological record of Fagaceae from the middle Eocene of western Greenland using a combined light microscopy (LM) and scanning electron microscopy (SEM) approach. The unambiguous recognition of modern lineages of Fagaceae is discussed in the context of the early evolutionary history of the family. The fossil evidence presented here is used to re-examine the concepts of the Arcto-Tertiary Element and the Arcto-Tertiary Geoflora.

For this study several sedimentary rock types from the Aamaruutissaa Formation on Hareøen were processed and checked for their pollen/spore content. Only the resinite-rich coal bed and associated mudstones/claystones in the lower part of the sedimentary rock sequence were productive and contained numerous well-preserved pollen/spores. The palynoflora from the resinite-rich coal bed is very diverse, composed of different spores of pteridophytes and pollen of various gymnosperms and angiosperms (F. Grímsson et al. pers. observ.).

This is the first major contribution on the palynoflora from Hareøen accompanied by a detailed geological introduction. Work in progress will focus on special gymnosperm and angiosperm taxa/lineages identified in the pollen spectra and their systematic and phytogeographic relations. In later reports the palynoflora will be used to revise the macroflora described partly by Heer (1868–1883) and Nathorst (1885). Also, based on the geological background presented here and the combination of pollen/spores and macrofossils the palaeoecology, vegetation, and climate in this part of Greenland during the middle Eocene will be established.

## Materials and methods

### Origin of samples and preparation

The sedimentary samples used for this study were collected on the island Hareøen, West Greenland. The samples originate from a resinite-rich coal bed (e.g. Steenstrup 1874) and were collected during the 1883 Swedish Greenland Expedition of A. E. Nordenskiöld. The plant fossils and sedimentary specimens from this expedition are housed in the collection of the Swedish Museum of Natural History, Stockholm. Samples from the sedimentary rocks were washed and dried and hand ground in a mortar with a pestle. The resulting powder was boiled in concentrated HCl for 5 min. After decanting most of the HCl liquid, the remainder was boiled for ca 10 min in HF. The solution was then transferred to 4 L beakers filled with water. After settling, the liquid was decanted and the remainder was boiled in HCl for 5 min. After cooling and settling most of the HCl was decanted, the remaining solution was centrifuged and the deposit washed 3–4 times with water. The sample then was acetolyzed, washed again with water, and centrifuged up to 4 times. The final remaining organic material was mixed with glycerine and stored in small sample tubes for microscopic studies. The fossil Fagaceae pollen grains were investigated both by LM and SEM, using the single grain technique (e.g. Zetter 1989). This technique has proved to be very useful particularly for fossil pollen (e.g. Grímsson et al. 2011a, b, 2012a, b, 2014; Grímsson and Zetter 2011) as the taxonomic resolution increases dramatically when individual pollen grains are investigated both with LM and SEM. Drops from the sample tubes were transferred to glass slides and single Fagaceae pollen grains were picked out using a preparation needle with a human nasal hair mounted on it. The grains were placed on a separate slide in fresh drops of glycerine for LM photography. The Fagaceae pollen grains then were transferred to SEM stubs using the preparation needle and were washed with drops of absolute ethanol to remove the remaining glycerine. The stubs were sputter coated with gold and the pollen photographed in a SEM (JEOL 6400). Approximately 600 alleged Fagaceae pollen grains were studied in LM; of these, ca 200 were also studied in SEM, and selected grains were then photographed using the SEM. Parts of the original sedimentary rocks, all sample tubes, and SEM stubs prepared during this study are stored in the collection of the Department of Palaeontology, University of Vienna, Austria.

### Phylogenetic framework

The phylogenetic framework used to interpret fossil pollen taxa relied on recent molecular phylogenetic studies on oaks using different nuclear data sets (Oh and Manos 2008; Denk and Grimm 2010; Hipp et al. 2014; Hubert et al. 2014). For clarity and convenience we use the traditional subfamilial names of Oersted (1871). Castaneoideae denote the grade formed by *Castanopsis*, *Castanea*, *Lithocarpus*, *Chrysolepis*, and *Notholithocarpus*. Fagoideae denote the genus *Fagus* (without *Nothofagus*), and Quercoideae the six infrageneric groups of *Quercus* as defined by Denk and Grimm (2009, 2010). *Colombobalanus, Formanodendron,* and *Trigonobalanus* are here addressed under the collective term ‘trigonobalanoids’. The extinct *Eotrigonobalanus* is treated separately.

## Results

### Stratigraphic and sedimentological observations

**Fig. 1:**
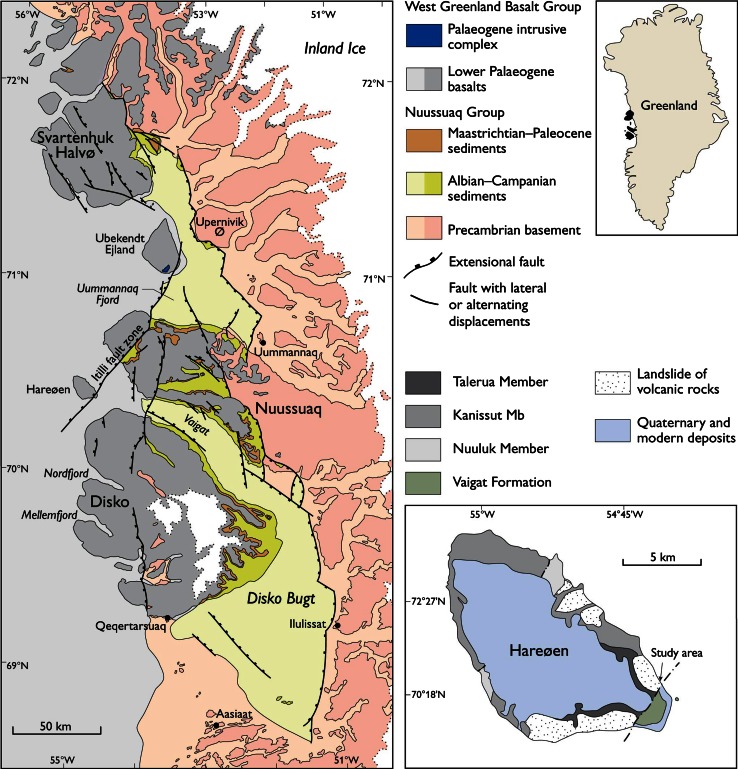
Geological maps modified after Dam et al. (2009) and Schmidt et al. (2005)

**Fig. 2:**
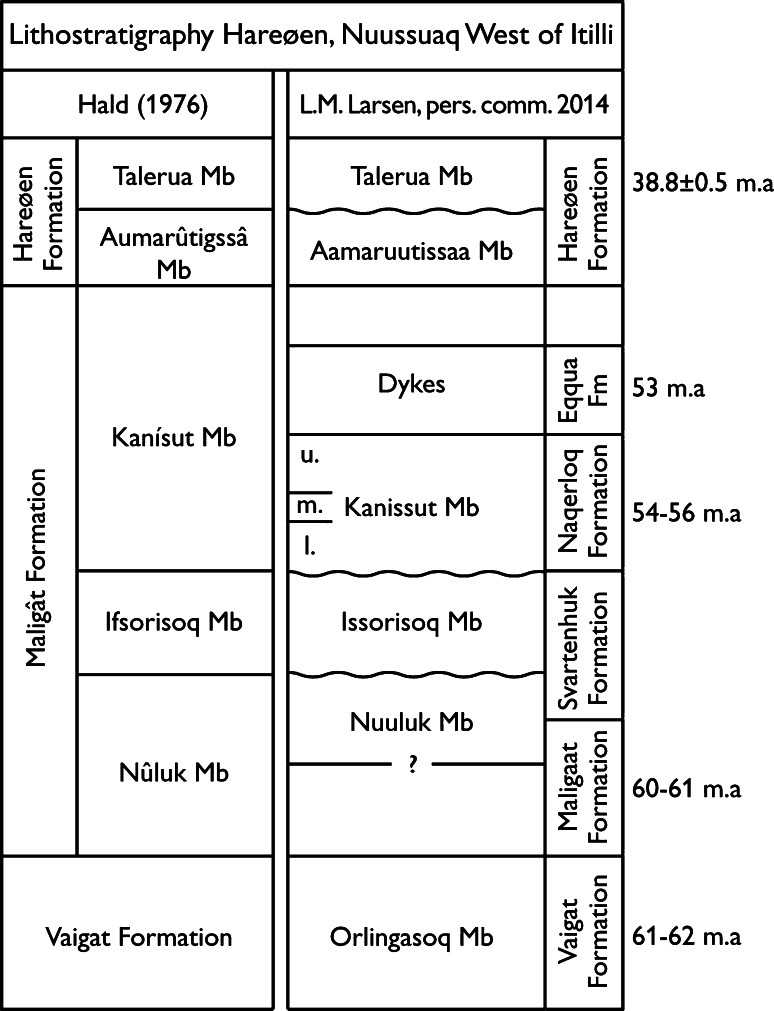
Lithostratigraphy of Hareøen and western Nuussuaq. The pioneering work by Hald (1976) is currently being revised (LM. Larsen pers. comm.). Radiometric ages from Larsen (pers. comm.), Storey et al. (1998), and Schmidt et al. (2005) are adjusted according to latest age determination of the Fish Canyon Tuff

The fossil Fagaceae pollen described here originate within the Nuussuaq Basin from sediments of the middle Eocene Aamaruutissaa Member of the Hareøen Formation, on the island Hareøen, West Greenland. The plant macrofossils from Hareøen described by Heer (1868–1883) and Nathorst (1885) also originate from the Aamaruutissaa Member. The Nuussuaq Basin is a fault-bounded basin on the west coast of Greenland that contains the only onshore exposures of Mesozoic and Cenozoic sedimentary rocks in West Greenland (Chalmers et al. 1999; Chalmers and Pulvertaft 2001; Oakey and Chalmers 2012; Gregersen et al. 2013). The Nuussuaq Basin comprises a succession of Cretaceous and early Paleocene siliciclastic sediments, referred to the Nuussuaq Group (Figs. 1, 2; Dam et al. 2009). The sediments are overlain by a thick series of volcanic rocks, the West Greenland Basalt Group, which also comprises intrabasaltic sedimentary units (Hald and Pedersen 1975). The island Hareøen provides the westernmost outcrops in the Nuussuaq Basin. The SE point of the island is cut by the NE-SW striking Itilli Fault Zone, which continues across western Nuussuaq (Chalmers et al. 1999; Fig. 1). It constitutes a part of the Ungava Fault Zone (Chalmers and Pulvertaft 2001). The central part of Hareøen is covered by a local icecap, which overlies a thick succession of Quaternary sediments. The Palaeogene of Hareøen comprises lava flows, dykes and sediments (Fig. 2). Hald (1976, 1977) established a lithostratigraphy of Hareøen and western Nuussuaq, including the Vaigat-, Maligât-, and Hareøen Formations (Fig. 2). The uppermost volcanic rocks in the Maligât Formation (sensu Hald 1977) are tholeiitic basalts, which were referred to the Kanísut Member (Fig. 2; Hald 1976). The overlying Hareøen Formation comprises arenaceous and argillaceous sediments with coal beds, the Aumarûtigssâ Member, and transitional basalts with phenocrysts of olivine, the Talerua Member (Hald 1976, 1977). Ongoing studies of the West Greenland Basalt Group are leading to a revision of the lithostratigraphy (Fig. 2; LM. Larsen pers. comm.).

**Fig. 3:**
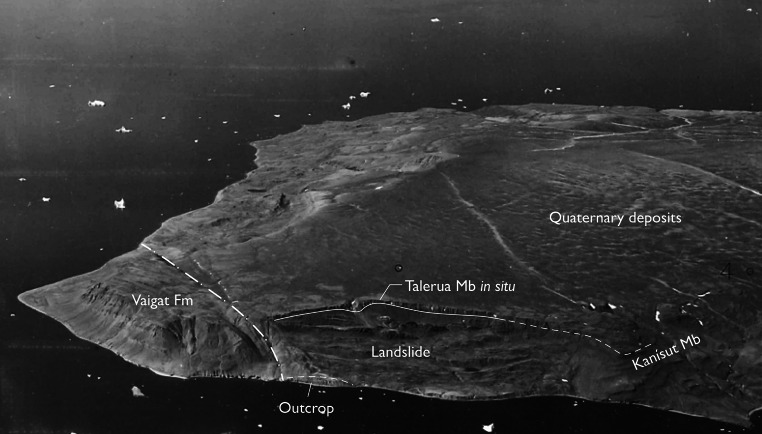
The Aamaruutissaa locality/outcrop in an oblique aerial photograph. The landslide is clearly seen, as well as the Talerua Member lava flows in situ at heights above 200 m a.s.l. North of the landslide the contact between lava flows of the Kanissut Member and the Talerua Member, respectively, has been described by Hald (1977)

The fossil pollen presented here derive from a resinite-rich coal bed in the intrabasaltic Aamaruutissaa Member (formerly spelled Aumarûtigssâ), which forms the lower part of the Hareøen Formation (Hald 1976, 1977). The Aamaruutissaa Member is only known from a few coastal exposures within the large landslide shown on Fig. 3. The Aamaruutissaa Member overlies the early Eocene Kanissut Member. The Kanissut Member belongs to the Naqerloq Formation (LM. Larsen pers. comm.), which is dated to 56–54 Ma (Storey et al. 1998; Dam et al. 2009). The Aamaruutissaa Member is overlain by the late Eocene Talerua Member (Hald 1976, 1977; LM. Larsen pers. comm.; Fig. 2). The Talerua Member lava flows have been dated radiometrically to 38.8 ± 0.5 Ma (Schmidt et al. 2005) suggesting that the underlying plant fossil bearing sediments of the Aamaruutissaa Member are slightly older. Our pollen analyses suggest a late Lutetian to early Bartonian (42–40 Ma) age for the plant bearing sediments.

**Fig. 4:**
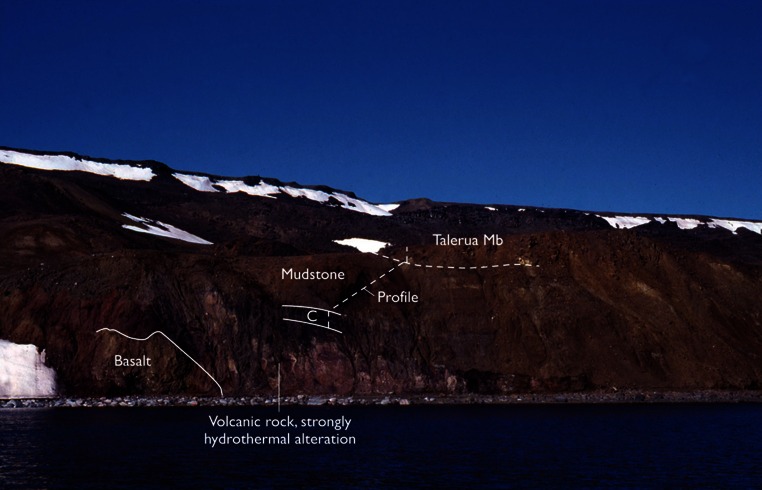
The Aamaruutissaa locality. The coal beds (C) and the overlying mudstones are indicated. The samples 422 002–016 were collected along the profile

The outcrop at Aamaruutissaa is found at sea level (Figs. 3, 4). The base of the outcrop is a red, strongly altered lava flow, which is veined by calcite in the form of silkspar. The lava flow is more than 5 m thick and composed of basalt believed to be part of the Kanissut Member. The lava is strongly eroded on top and covered by several metres of reddish clay mixed with decimetre sized bodies of fine-grained white very strongly decomposed volcanic material. The altered volcanic rocks are soft, and their dark purple, pink and almost white colours indicate that some or most of the original minerals are altered to clays. This is confirmed by the X-ray diffraction analyses. Comparable alterations are not seen in the overlying sediments, which suggest that the alterations preceded deposition of sediments of the Aamaruutissaa Member. The sediments of the Aamaruutissaa Member are considered of terrestrial freshwater origin (non-marine). The sediments in the outcrop are overlain by a columnar jointed basalt lava flow of the Talerua Member (Hareøen Formation). Above the landslide lava flows of the Talerua Member are observed in situ at a height of about 200 m (Rosenkrantz et al. 1976; Schmidt et al. 2005; Figs. 3, 4).

**Fig. 5:**
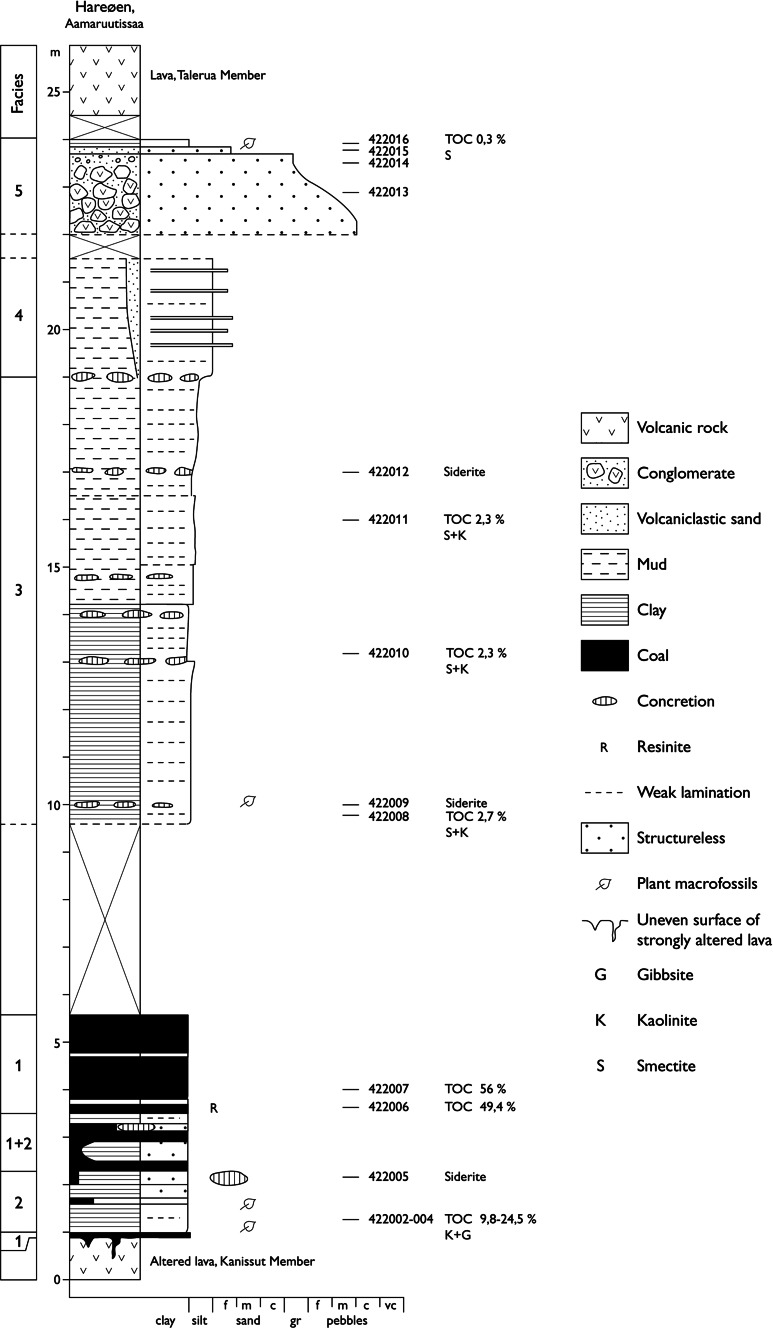
Sedimentological log from the Aamaruutissaa locality, see also Fig. 4. The 24-m thick succession is divided into five sedimentary facies. Samples collected during field work numbered 422 002–422 016 are indicated. Some of the samples have been analysed for mineralogy and TOC content

The sedimentary succession at the Aamaruutissaa locality is about 24 m thick and composed of five distinguished facies (Figs. 4, 5). Facies 1: The coal beds are 0.2–0.9 m thick and interbedded with mudstone. Pyrolysis screening analyses of two coal samples show TOC contents of 50–60 %, which indicate a significant content of clay. The analyses of few mudstone samples show low Tmax values (415–430 °C), which suggest low maturity. The coal samples have a high potential for generating hydrocarbons (high S2 values), and the hydrogen index (HI) values are above 100, which suggest that degradation was only moderate. The coal is dark brown to black, locally with high concentrations of resinite (retinite), which occurs in 0.5- to 1-cm thick laminae. The resinite occurs as spherical accumulations which are about 5 mm in diameter and somewhat irregular in shape and showing no signs of transport and reworking. Pieces of coalified wood occur locally in the coal. The clay content in the coal beds and the mudstones, interbedded with the coal beds, indicates deposition in a low-energy environment, such as a lake or pond, which continually received clay in suspension. Organic geochemistry shows that the coal beds of Aamaruutissaa have never been subjected to elevated temperatures, neither during accumulation of plant remains nor subsequently. Facies 2: Greyish brown to black and weakly consolidated claystones that are structureless. The claystones are interbedded with the coal beds, and frequently rich in coalified plant macrofossils. The colour of the claystones ranges due to varying contents of comminuted plant debris. This is reflected in a high TOC content, 9.8–24.5 %, in samples 422 002–422 004 (Fig. 5). The claystones contain siderite concretions. The abundance of plant remains, the presence of siderite, and the absence of sandstone beds indicate that the claystones were deposited in a low-energy lake or pond.

Facies 3: Olive grey and structureless or weakly laminated claystones are rich in small plant fragments. The TOC content is 2.3 % (samples 422 010/422 011). Certain horizons in the claystones contain brownish siderite concretions, which are about 5 cm thick and about 25 cm in diameter (Fig. 5). Upwards, the claystones become slightly silty and the colour changes to brown. Silt (and sand) constitutes 15–70 % of the samples. Quartz has not been detected, the lack of quartz shows that the sediments comprise only local material derived from the adjacent weathered lava flows. Facies 4: Brownish silty claystones with laminae of sandstones. The silty clays show small variations in colour. The laminae of fine-grained sandstones are 2–10 mm thick, without sedimentary structures, and the particles are of volcanic glass. The presence of volcanic ash indicates beginning volcanic activity, which preceded the eruption of the Talerua Member lava flows. Facies 5: Conglomerate of rounded, well-sorted pebble-size clasts of basaltic to basalto-andesitic volcanic rocks. Several of the volcanic clasts have compositions that are not known from any lava successions within the Nuussuaq Basin. Some of the clasts originate from the Talerua Member, which overlies the sedimentary succession. This indicates that the conglomerate is contemporaneous with the lower part of the Talerua Member. The conglomerate is clast supported with a matrix of volcaniclastic sand. The colour of the conglomerate and the overlying thin sandstone and mudstone is pale green, almost white, and TOC is likewise low, 0.3 % (Fig. 5). A thin bed of abundant plant remains, mostly stems, twigs and leaves, is interbedded in the sandstone in the top of the conglomerate. The plant fossils appear to have been transported by and deposited from currents. The sorted and rounded clasts indicate transport in running water, probably a stream, which carried clasts from a varied source area. Decreasing current velocity is indicated by the normal grading in the top of the conglomerate. The conglomerate is overlain by a single lava flow of the Talerua Member.

### Palaeoenvironment and origin of plant remains

Geological observations suggest that the sediments of the Aamaruutissaa Member are localised and accumulated in a small depression outlined by lavas of the Kanissut Member. The sediments accumulated in a shallow freshwater lake environment. Our palynological investigations suggest that the terrestrial palynoflora from the basal units of the sedimentary sequence is between 42–40 Ma and the overlying basalts have been dated to ca. 39 Ma (see above). This indicates that the lake sediments might have accumulated over a period of 1–3 million years. All the sediments below facies 5 suggest a low-energy shallow lake environment and the high content of plant debris within the fine-grained sedimentary units indicates that the lake margins and associated streams were covered with vegetation. This is also supported by the high number of pollen and spores representing aquatic to wetland plants and plants thriving in backswamp forests and in temporally flooded or well-drained lake margin areas (F. Grímsson et al. unpubl. data). The excellent preservation status of the pollen grains suggests that most of them originate from close vicinity to the lake. Numerous pollen clumps are found in the samples and many of the pollen still have Ubisch bodies suggesting that the grains were transported within a flower/anther into the sediments. Therefore, the palynoflora and the Fagaceae pollen presented here are believed to originate from plants/trees growing in a forest reaching the lake margin.

### Systematic Palaeobotany

The descriptions of Fagaceae pollen grains include diagnostic features observed under LM and SEM. The terminology for description follows Punt et al. (2007) and Hesse et al. (2009).

**Family Fagaceae** Dumort.

**Genus** ***Eotrigonobalanus*** Walther and Kvaček (extinct)

**Fig. 6:**
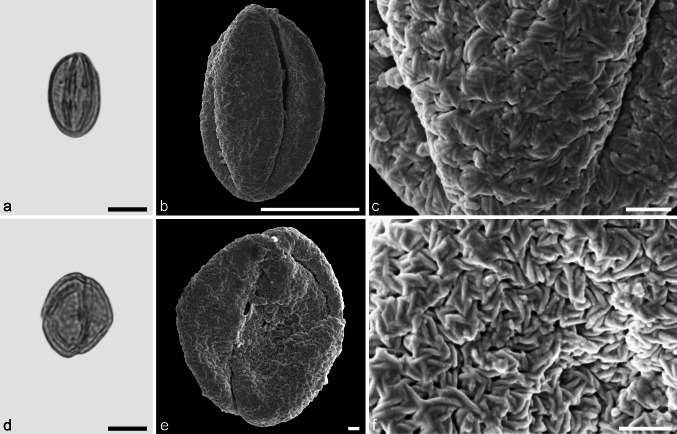
Fossil Fagaceae pollen from the Eocene of West Greenland. **a** and **d** LM micrographs. **b**, **c**, **e**, and **f** SEM micrographs. All pollen grains are shown in equatorial view. **a**–**f** *Eotrigonobalanus* sp. *Scale bar* is 10 µm in **a**, **b**, and **d**, and 1 µm in **c**, **e**, and **f**

***Eotrigonobalanus*** **sp.** (Fig. 6a–f)

*Description.* Pollen, monad, prolate, circular to lobate in polar view, elliptic in equatorial view; polar axis 21–23 µm long in LM, 18–21 µm long in SEM, equatorial diameter 14–18 µm wide in LM, 13–15 µm wide in SEM; tricolporate, colpi long, endopori elongated rectangular, nexine slightly thickened around endopori (LM); exine 1.1–1.2 µm thick, nexine slightly thinner than sexine; tectate; sculpturing scabrate in LM, rugulate, perforate in SEM, rugulae twisted and interwoven, forming larger rugulae that encircle perforations (SEM).

*Remarks.* The fossil *Eotrigonobalanus* pollen grains from Hareøen show a high variability in size, shape and arrangement of sculpturing elements. Similar variation has been noticed in *Eotrigonobalanus eiszmannii* Walther & Kvaček pollen from the early Oligocene of Cospuden, Germany (Denk et al. 2012), and in fossil pollen grains from clumps attached to the leaf surface of *Eotrigonobalanus* *furcinervis* from Oligocene sediments of Witznitz, Germany (Walther and Zetter 1993). Fossil pollen grains that are similar to the grains from Hareøen have been described from the Paleocene/Eocene boundary of Salzburg, Austria (*Eotrigonobalanus*; Hofmann 2010; Hofmann et al. 2011), from the middle to late Oligocene of Texas (*Amentoplexipollenites*; Crepet and Nixon 1989), and from middle Miocene sediments of Poland (Kohlman-Adamska and Ziembińska-Tworzydło 2000; Stuchlik et al. 2007). This is the first report of *Eotrigonobalanus* pollen from Greenland.

**Subfamily Castaneoideae** Oerst.

Pollen morphology of Castaneoideae has been thoroughly studied using LM, SEM and TEM (e.g. Praglowski 1984). The pollen of *Castanea*, *Castanopsis*, *Chrysolepis, Lithocarpus,* and *Notholithocarpus* are very similar in size and shape. Using LM only, they are indistinguishable at the generic level. Pollen of *Castanopsis* and *Lithocarpus/Notholithocarpus* commonly overlap in size and shape and arrangement of sculpturing elements seen under SEM, and cannot be distinguished. *Castanea* type pollen are generally smaller and narrower than pollen of the other genera. Under the SEM the rugulate sculpturing is flattened and smoother (fused) in appearance (Praglowski 1984).

**Fig. 7:**
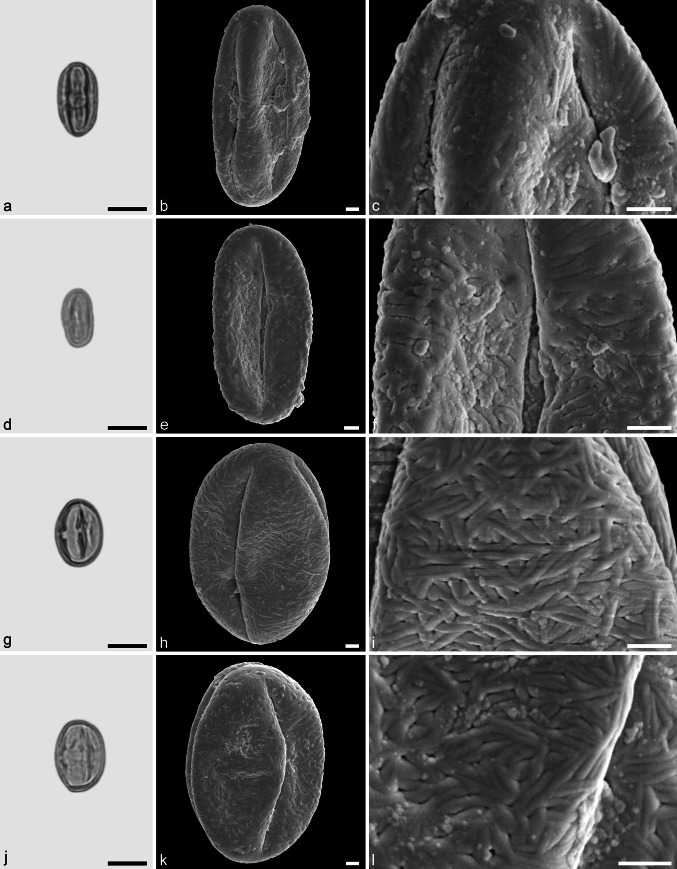
Fossil Castaneoideae pollen from the Eocene of West Greenland. **a**, **d**, **g**, and **j** LM micrographs. **b**, **c**, **e**, **f**, **h**, **i**, **k**, and **l** SEM micrographs. All pollen grains are shown in equatorial view. **a**–**f** Castaneoideae sp. 1 (aff. *Castanea*) **g–l** Castaneoideae sp. 2 (aff. *Castanopsis*). *Scale bar* is 10 µm in **a**, **d**, **g**, and **j**, and 1 µm in **b**, **c**, **e**, **f**, **h**, **i**, **k**, and **l**

Castaneoideae sp. 1 (aff. *Castanea*) (Fig. 7a–f)

*Description.* Pollen, monad, prolate, outline lobate in polar view, elliptic in equatorial view; polar axis 16–20 µm long in LM, 14–18 µm long in SEM, equatorial diameter 8–11 µm wide in LM, 7–9 µm wide in SEM; tricolporate, pori lalongate, colpi long; exine 0.9–1.0 µm thick (LM), nexine thinner than sexine; tectate; sculpturing psilate in LM, rugulate, fossulate, perforate in SEM.

*Remarks.* Castaneoideae sp. 1 pollen from Hareøen has short and broad rugulae that are indistinct. The rugulae usually are fused together and show little relief, especially in the polar areas. This is typical for pollen of many extant *Castanea* species, and may be taken as an indication that Castaneoideae sp. 1 is a representative of this genus.

Castaneoideae sp. 2 (aff. *Castanopis*) (Fig. 7g–l)

*Description.* Pollen, monad, prolate, outline lobate in polar view, elliptic in equatorial view; polar axis 17–20 µm long in LM, 15–18 µm long in SEM, equatorial diameter 13–14 µm wide in LM, 10–12 µm wide in SEM; tricolporate, pori lalongate, colpi long; exine 0.9–1.0 µm thick (LM), nexine thinner than sexine; tectate; sculpturing psilate in LM, rugulate, fossulate, perforate in SEM; rugulae fused and showing a weak relief in polar areas; parallel running individual rugulae occasionally arranged in bundles.

*Remarks*: The occasional organisation of individual rugulae in bundles in Castaneoideae is only rarely documented in the literature. Praglowski (1984) figured pollen of *Castanopsis cuspidata* (Thunb.) Schottky showing this feature. However, there seems to be a certain variation at the species level (Miyoshi 1983; J. Bouchal et al., unpublished data). To our knowledge, this feature has not been reported for other genera in Castaneoideae.

**Fig. 8:**
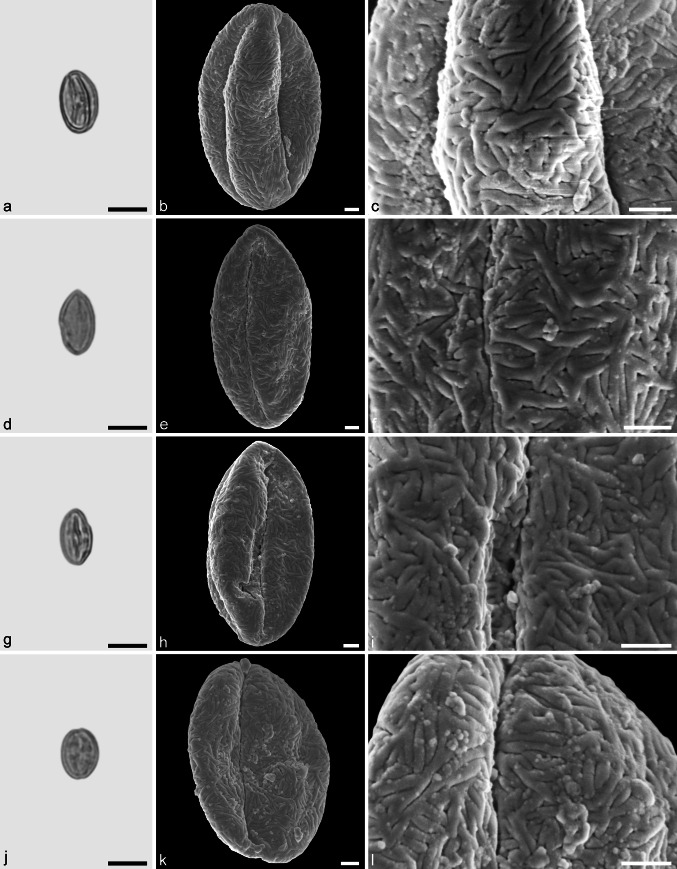
Fossil Castaneoideae pollen from the Eocene of West Greenland. **a**, **d**, **g**, and **j** LM micrographs. **b**, **c**, **e**, **f**, **h**, **i**, **k**, and **l** SEM micrographs. All pollen grains are shown in equatorial view. **a**–**l** Castaneoideae sp. 3. *Scale bar* is 10 µm in **a**, **d**, **g**, and **j**, and 1 µm in **b**, **c**, **e**, **f**, **h**, **i**, **k**, and **l**

Castaneoideae sp. 3 (Fig. 8)

*Description.* Pollen, monad, prolate, outline lobate in polar view, elliptic in equatorial view; polar axis 14–18 µm long in LM, 13–15 µm long in SEM, equatorial diameter 9–10 µm wide in LM, 7–9 µm wide in SEM; tricolporate, pori lalongate, colpi long; exine 0.8–0.9 µm thick (LM), nexine thinner than sexine; tectate; sculpturing psilate in LM, rugulate, fossulate, perforate in SEM; rugulae equally developed across the entire pollen grain; individual rugulae not organised in bundles; rugulae in the polar areas often running parallel to the polar axis.

*Remarks.* This pollen type is very common among all modern genera (except perhaps *Castanea*) of Castaneoideae and cannot be assigned to a particular genus.

**Subfamily Fagoideae** K.Koch

**Genus** ***Fagus*** L.

**Subgenus** ***Fagus***

**Fig. 9:**
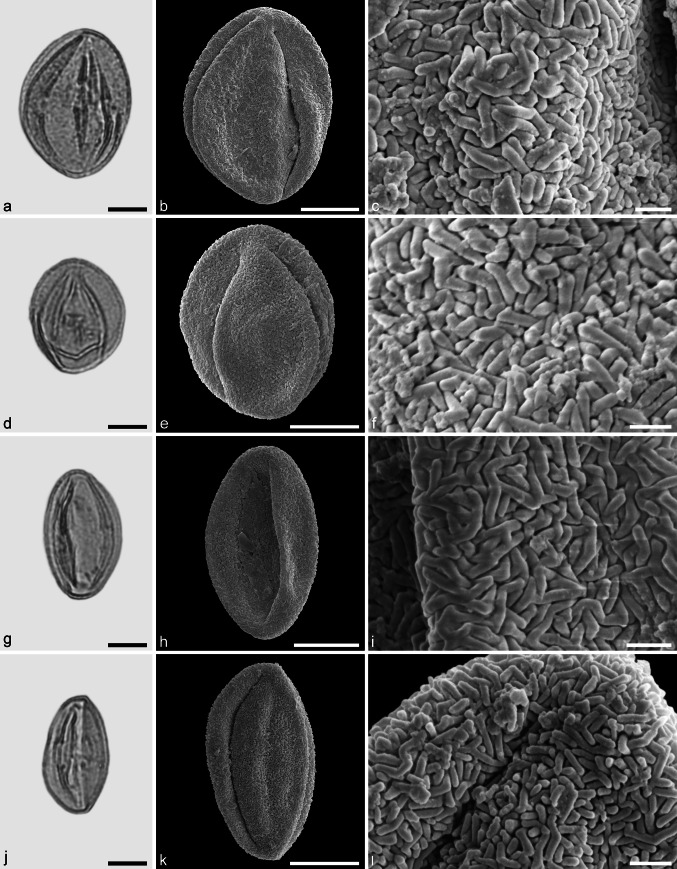
Fossil Fagoideae pollen from the Eocene of West Greenland. **a**, **d**, **g**, and **j** LM micrographs. **b**, **c**, **e**, **f**, **h**, **i**, **k**, and **l** SEM micrographs. All pollen grains are shown in equatorial view. **a**–**l** *Fagus* sp. *Scale bar* is 10 µm in **a**, **b**, **d**, **e**, **g**, **h**, **j**, and **k**, and 1 µm in **c**, **f**, **i**, and **l**

**Fig. 10:**
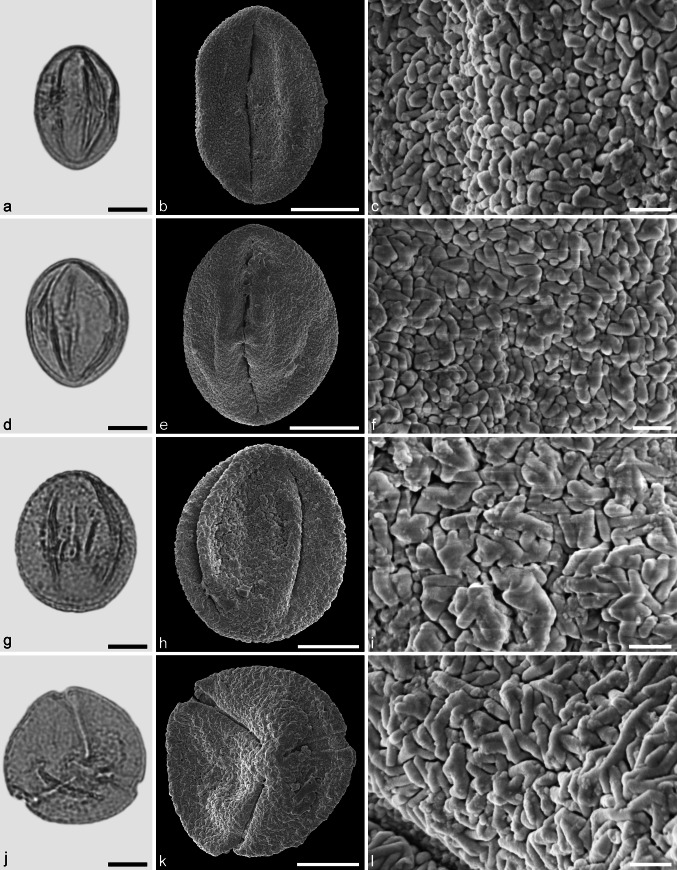
Fossil Fagoideae pollen from the Eocene of West Greenland. **a**, **d**, **g**, and **j** LM micrographs. **b**, **c**, **e**, **f**, **h**, **i**, **k**, and **l** SEM micrographs. Most pollen grains are shown in equatorial view, **j**–**l** in polar view. **a**–**l** *Fagus* sp. *Scale bar* is 10 µm in **a**, **b**, **d**, **e**, **g**, **h**, **j**, and **k**, and 1 µm in **c**, **f**, **i**, and **l**

***Fagus*** **sp.** (Figs. 9, 10)

*Description.* Pollen, monad, spheroidal, outline circular in polar and equatorial views; polar axis 32–38 µm long in LM, 28–35 µm long in LM, equatorial diameter 18–38 µm wide in LM, 16–29 µm wide in SEM; tricolporate, nexine slightly thickened around pori; exine 0.8–1.2 µm thick (LM), nexine thinner than sexine; tectate; sculpturing scabrate in LM, rugulate and fossulate in SEM, rugulae often diverging and protruding, occasionally fused forming distinct protruding clusters (SEM).

*Remarks.* *Fagus* pollen of extant species have been studied using LM, SEM, and TEM. Pollen of the different species are very similar and most of them overlap in morphology. Species of the subgenus *Engleriana* differ by narrow colpi that usually reach the poles and the grains are smaller than in the remaining species (e.g. Praglowski 1982; Denk 2003). The size of the fossil pollen and the arrangement of the colpi suggest that they belong to subgenus *Fagus*.

*Fagus* pollen grains are frequent in the Hareøen samples and show a high variability in form, size, and sculpturing. Many of the grains have short and broad and undivided rugulae (see Figs. 9f, l, 10c, f). Some pollen grains have fused and robust clustered rugulae (Fig. 10i). These types of pollen grains may have entered the sediments while still in the flower/anther. This has also been observed in extant flower material of *Fagus* (R. Zetter pers. observ.) and in in situ herbarium material (T. Denk pers. observ., RP Farges 15-57B [E]). This is the first record of *Fagus* pollen from the Eocene of Greenland.

Subfamily Quercoideae Oerst.

**Genus** ***Quercus*** L.

Comprehensive studies on the pollen morphology of extant *Quercus* species have shown that SEM sculpturing can be used to distinguish between major infrageneric groups of the genus (Solomon 1983a, 1983b; Denk and Grimm 2009; Denk and Tekleva 2014).

**Fig. 11:**
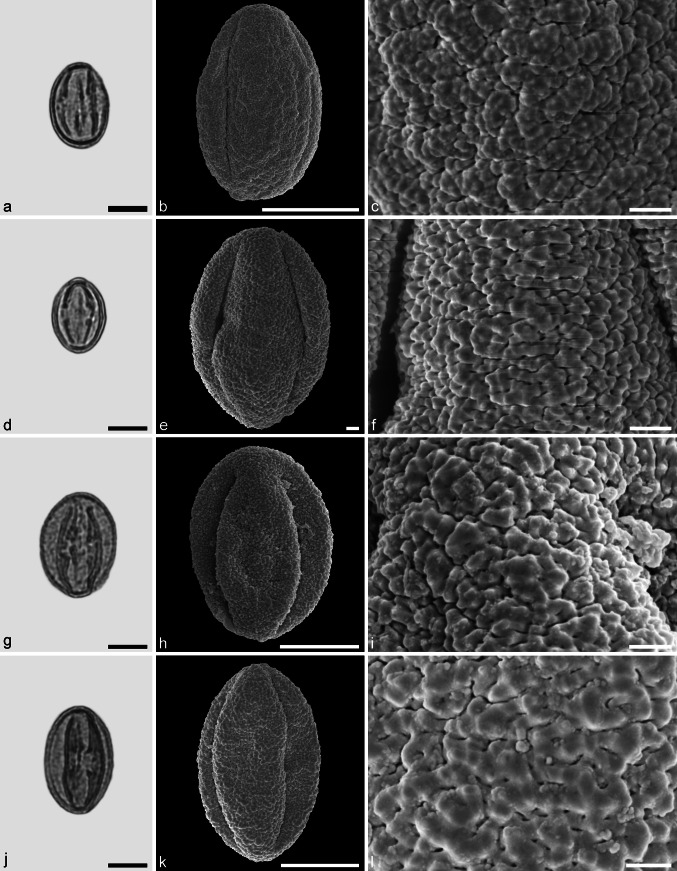
Fossil Quercoideae pollen from the Eocene of West Greenland. **a**, **d**, **g**, and **j** LM micrographs. **b**, **c**, **e**, **f**, **h**, **i**, **k**, and **l** SEM micrographs. All pollen grains are shown in equatorial view. **a**–**l** *Quercus* sp. 1 (aff. Group Lobatae). *Scale bar* is 10 µm in **a**, **b**, **d**, **g**, **h**, **j**, and **k**, and 1 µm in **c**, **e**, **f**, **i**, and **l**

*Quercus* sp. 1 (aff. Group Lobatae) (Fig. 11)

*Description.* Pollen, monad, prolate, outline lobate in polar view, elliptic in equatorial view; polar axis 21–29 µm long in LM, 15–26 µm long in SEM, equatorial diameter 14–20 µm wide in LM, 11–19 µm wide in SEM; tricolporate, nexine thickened around the endopori (LM); exine 1.2–1.5 µm thick (LM), nexine thinner than sexine; tectate; sculpturing scabrate in LM, microverrucate/verrucate to microrugulate/rugulate, fossulate, perforate in SEM, verrucae and rugulae with a microechinate suprasculpture, microechini often poorly developed, irregularly distributed (SEM).

*Remarks.* This pollen type is closely similar to a number of species of *Quercus* Group Lobatae of North America (Solomon 1983b).

**Fig. 12:**
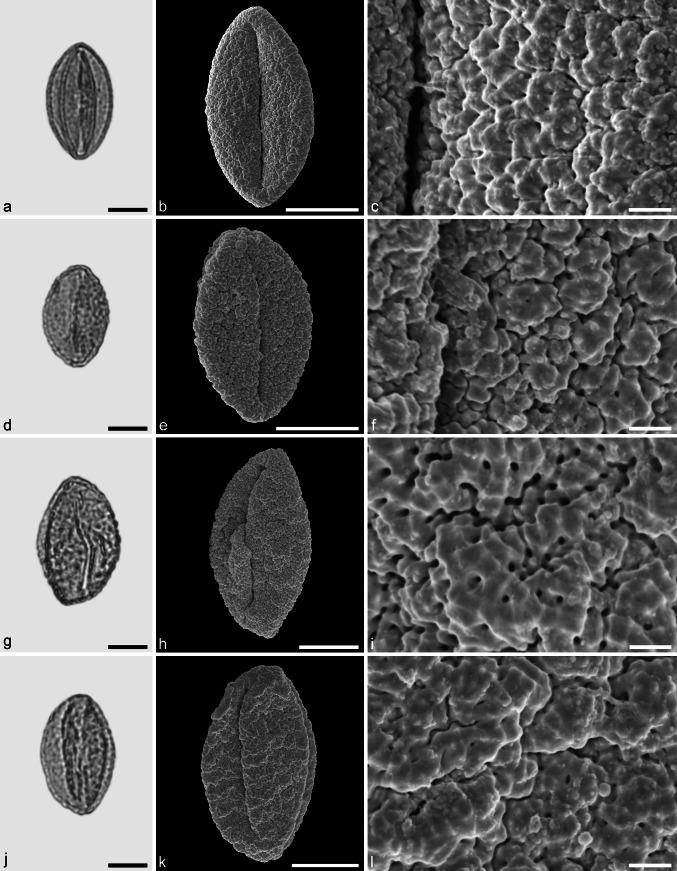
Fossil Quercoideae pollen from the Eocene of West Greenland. **a**, **d**, **g**, and **j** LM micrographs. **b**, **c**, **e**, **f**, **h**, **i**, **k**, and **l** SEM micrographs. All pollen grains are shown in equatorial view. **a**–**l** *Quercus* sp. 2 (aff. Group Lobatae). *Scale bar* is 10 µm in **a**, **b**, **d**, **e**, **g**, **h**, **j**, and **k**, and 1 µm in **c**, **f**, **i**, and **l**

*Quercus* sp. 2 (aff. Group Lobatae) (Fig. 12)

*Description.* Pollen, monad, prolate, outline lobate in polar view, elliptic in equatorial view; polar axis 28–34 µm long in LM, 24–32 µm long in SEM, equatorial diameter 18–22 µm wide in LM, 14–18 µm wide in SEM; tricolporoidate; exine 0.8–1.2 µm thick (LM), nexine thinner than sexine; tectate; sculpturing scabrate in LM, verrucate to rugulate, fossulate, perforate in SEM, verrucae and rugulae with a microechinate suprasculpture, microechini occurring on narrow ridges (SEM).

*Remarks.* This type of pollen differs from the *Quercus* sp. 1 pollen type as it has no clear pori (poroidate) and no prominent thickenings around the pori (cf. Figs. 11, 12). Also, the sculpturing of *Quercus* sp. 2 is more robust and shows a more prominent relief than in *Quercus* sp. 1. This pollen type is closely similar to a number of species of *Quercus* Group Lobatae of North America (Solomon 1983b).

**Fig. 13:**
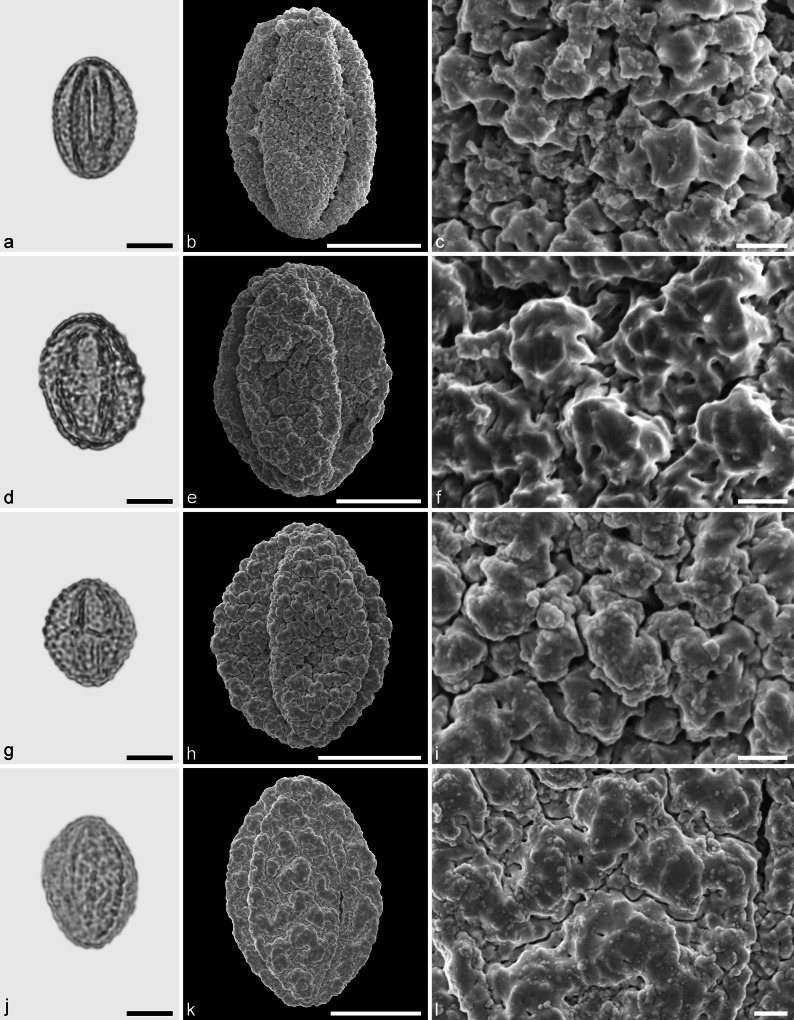
Fossil Quercoideae pollen from the Eocene of West Greenland. **a**, **d**, **g**, and **j** LM micrographs. **b**, **c**, **e**, **f**, **h**, **i**, **k**, and **l** SEM micrographs. All pollen grains are shown in equatorial view. **a**–**l** *Quercus* sp. 3 (Group Lobatae vel Group Quercus). *Scale bar* is 10 µm in **a**, **b**, **d**, **e**, **g**, **h**, **j**, and **k**, and 1 µm in **c**, **f**, **i**, and **l**

*Quercus* sp. 3 (Group Lobatae vel Group Quercus) (Fig. 13)

*Description.* Pollen, monad, prolate, outline lobate in polar view, elliptic in equatorial view; polar axis 25–33 µm long in LM, 21–28 µm long in SEM, equatorial diameter 19–23 µm wide in LM, 16–18 µm wide in SEM; tricolporoidate; exine 1.3–1.6 µm thick (LM), nexine thinner than sexine; tectate; sculpturing scabrate to verrucate in LM, verrucate to rugulate, perforate in SEM, verrucae and rugulae with a microechinate suprasculpture (SEM).

*Remarks.* This pollen type differs from the *Quercus* sp. 1 and sp. 2 by its sculpturing. *Quercus* sp. 3 pollen has much more prominent and protruding verrucae and rugulae and distinctive grooves between the verrucae and rugulae. The verrucate sculpturing also shows much less perforations. Such pollen types are encountered in modern members of *Quercus* Group Lobatae and Group Quercus (Solomon 1983a, b).

**Fig. 14:**
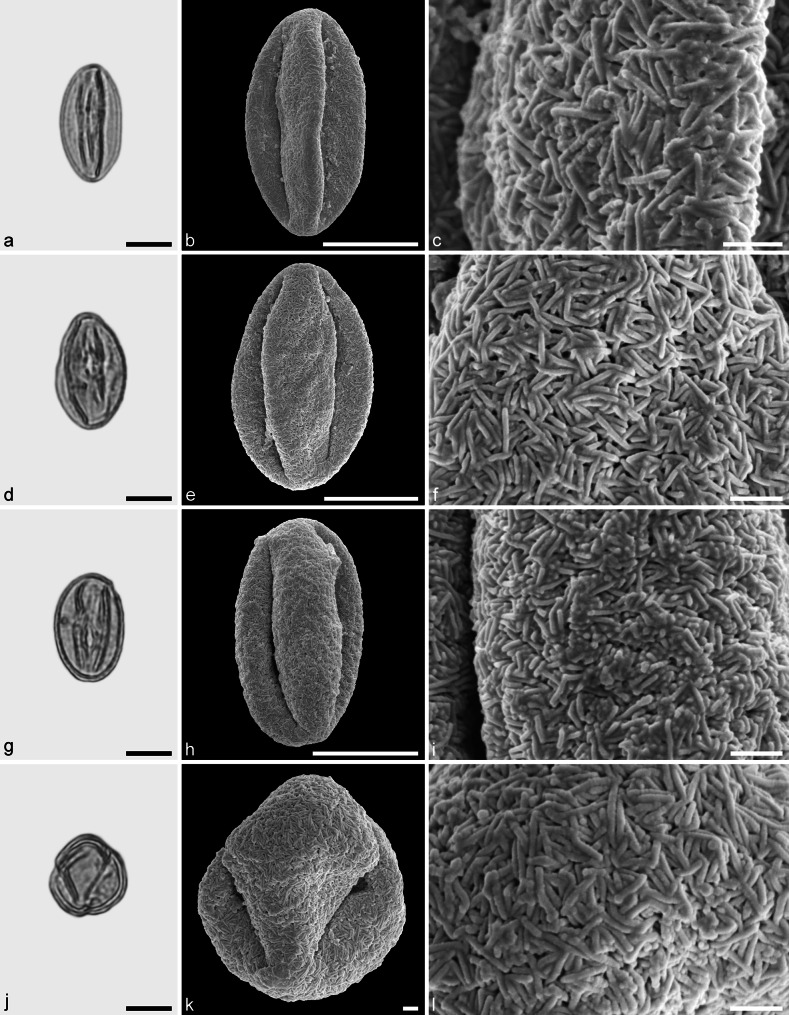
Fossil Quercoideae pollen from the Eocene of West Greenland. **a**, **d**, **g**, and **j** LM micrographs. **b**, **c**, **e**, **f**, **h**, **i**, **k**, and **l** SEM micrographs. All pollen grains are shown in equatorial view. **a**–**l** *Quercus* sp. 4 (putative ancestral lineage with Group Ilex morphology). *Scale bar* is 10 µm in **a**, **b**, **d**, **e**, **g**, **h**, and **j**, and 1 µm in **c**, **f**, **i**, **k**, and **l**

*Quercus* sp. 4 (putative ancestral lineage with Group Ilex morphology) (Fig. 14)

*Description.* Pollen, monad, prolate, outline lobate in polar view, elliptic in equatorial view; polar axis 20–26 µm long in LM, 18–24 µm long in SEM, equatorial diameter 14–18 µm wide in LM, 12–15 µm wide in SEM; tricolporoidate; exine 0.9–1.1 µm thick (LM), nexine thinner than sexine; tectate; sculpturing scabrate in LM, rugulate, perforate in SEM, rugulae are narrow and rod-like in form, irregularly arranged (SEM).

*Remarks.* This fossil pollen type shows considerable variability in form, size and arrangement of the rod-like rugulae. All the sculpturing forms of this pollen type are corresponding to the ornamentation reported for extant pollen of *Quercus* Group Ilex (Denk and Grimm 2009; Denk and Tekleva 2014). Denk and Grimm (2009) speculated that the Group Ilex pattern of pollen sculpturing may be the plesiomorphic state for *Quercus*. If this is true, this pollen sculpturing might also be encountered in extinct lineages of *Quercus*. At present, *Quercus* Group Ilex (subgenus *Heterobalanus*) has a distribution range in Eurasia from Northwestern Africa and Southwestern Europe to the southern foothills of the Himalayas, western China and into Japan and Southeast Asia (Menitsky 2005). The group has no fossil and modern record in North America. The present finding is unexpected and, in the absence of leaf types that can be linked to the pollen, difficult to interpret.

**Fig. 15:**
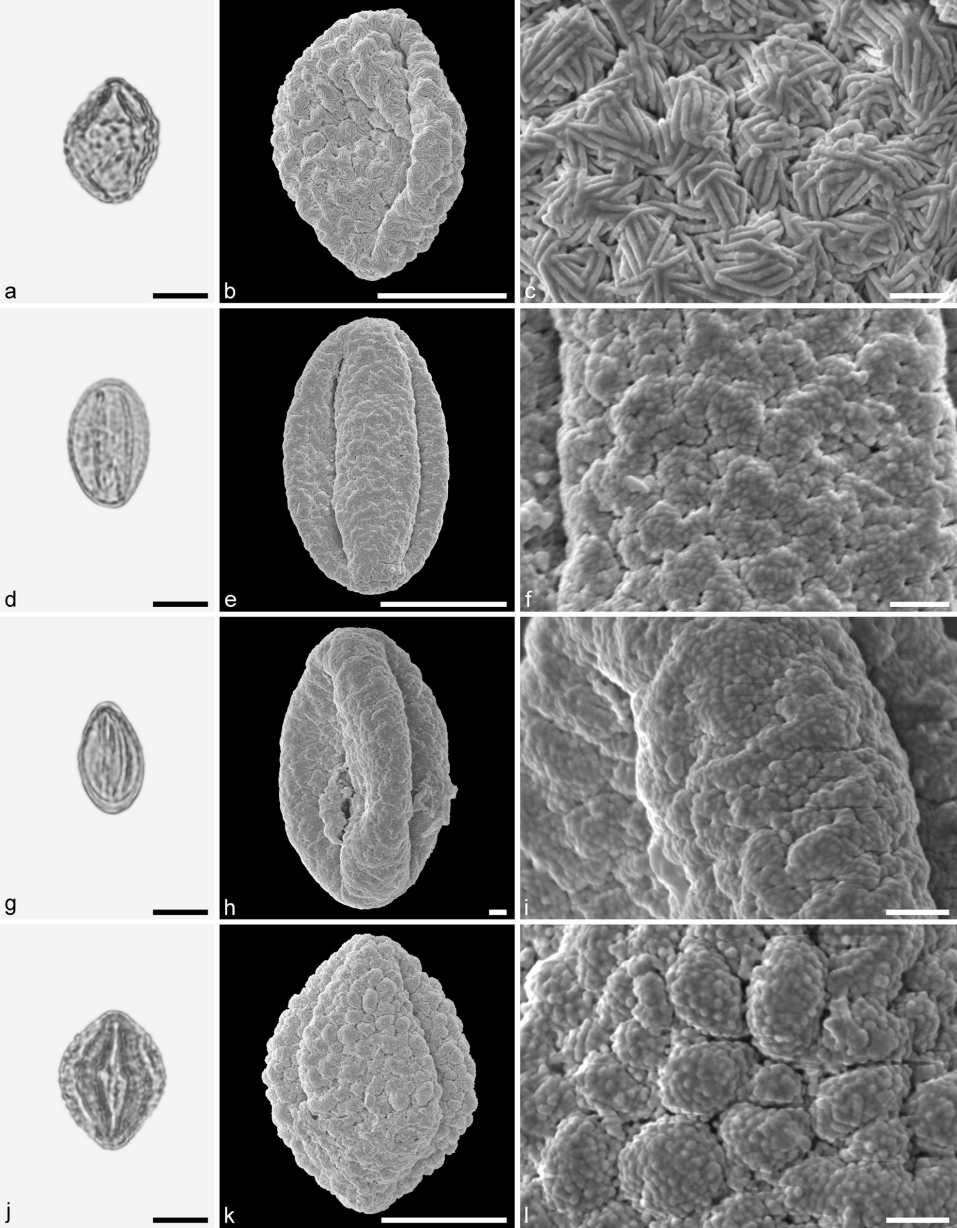
Fossil Quercoideae pollen from the Eocene of West Greenland. **a**, **d**, **g**, and **j** LM micrographs. **b**, **c**, **e**, **f**, **h**, **i**, **k**, and **l** SEM micrographs. All pollen grains are shown in equatorial view. **a**–**c** *Quercus* sp. 5 (ancestral or extinct lineage). **d**–**i** *Quercus* sp. 6 (aff. Group Protobalanus). **j**–**l** *Quercus* sp. 7 (aff. Group Protobalanus). *Scale bar* is 10 µm in **a**, **b**, **d**, **e**, **g**, **j**, and **k**, and 1 µm in **c**, **f**, **h**, **i**, and **l**

*Quercus* sp. 5 (ancestral or extinct lineage) (Fig. 15a–c)

*Description.* Pollen, monad, prolate, outline lobate in polar view, elliptic in equatorial view; polar axis 23–25 µm long in LM, 21–23 µm long in SEM, equatorial diameter 17–19 µm wide in LM, 15–17 µm wide in SEM; tricolporate; exine 1.6–1.8 µm thick (LM), nexine thinner or as thick as sexine; tectate; sculpturing verrucate in LM and SEM, verrucae composed of narrow rod-like rugulae, rugulae in groups, groups oriented (SEM).

*Remarks.* This fossil pollen type is considered to represent an extinct taxon/lineage within *Quercus*. It shows the same basic plesiomorphic sculpturing under SEM as is known from Group Ilex (see Fig. 1 in Denk and Grimm 2009). In contrast to all modern members of Group Ilex (Denk and Tekleva 2014), the number of parallel arranged rugulae forming desert rose-like clusters is consistently three or more. Clusters of rugulae form a more distinctly verrucate sculpture than encountered in modern Ilex oaks.

*Quercus* sp. 6 (aff. Group Protobalanus) (Fig. 15d–i)

*Description.* Pollen, monad, prolate, outline lobate in polar view, elliptic in equatorial view; polar axis 22–25 µm long in LM, 16–22 µm long in SEM, equatorial diameter 13–15 µm wide in LM, 10–13 µm wide in SEM; tricolporoidate; exine 0.9–1.0 µm thick (LM), nexine thinner than sexine; tectate; sculpturing scabrate to verrucate in LM, verrucate to rugulate, fossulate, perforate in SEM, verrucae and rugulae with a microrugulate suprasculpture (SEM).

*Remarks.* The sculpturing of the *Quercus* sp. 6 pollen type is similar to what has been reported for pollen grains from extant species of the Group Protobalanus (see Fig. 5c–e in Denk and Grimm 2009). Today *Quercus* Group Protobalanus is only represented by five species thriving under a Mediterranean climate (Csa, Csb climates) in western North America. Moreover, the pollen type of Group Protobalanus can be considered to be plesiomorphic for Group Lobatae/Quercus. It is hence impossible to judge based merely on pollen morphology whether the fossil pollen represents a member of Group Protobalanus or an ancestral/extinct lineage of the Protobalanus-Quercus-Lobatae clade (‘New World Clade’ according Manos et al. 2001).

*Quercus* sp. 7 (aff. Group Protobalanus) (Fig. 15j–l)

*Description.* Pollen, monad, prolate, outline lobate in polar view, elliptic in equatorial view; polar axis 26–28 µm long in LM, 21–23 µm long in SEM, equatorial diameter 19–21 µm wide in LM, 17–19 µm wide in SEM; tricolporate; exine 1.4–1.6 µm thick (LM), nexine thinner than sexine; tectate; sculpturing verrucate in LM, verrucate, fossulate in SEM, verrucae with a densely packed microechinate/microverrucate suprasculpture (SEM).

*Remarks.* Like *Quercus* sp. 6, this fossil pollen shows the basic sculpturing under SEM as is known from modern members of *Quercus* Group *Protobalanus* (see Fig. 5d in Denk and Grimm 2009), but the verrucae are more prominent than what has been reported for extant species.

## Discussion

### Temporal and phylogenetic framework of Fagaceae of western Greenland

**Fig. 16:**
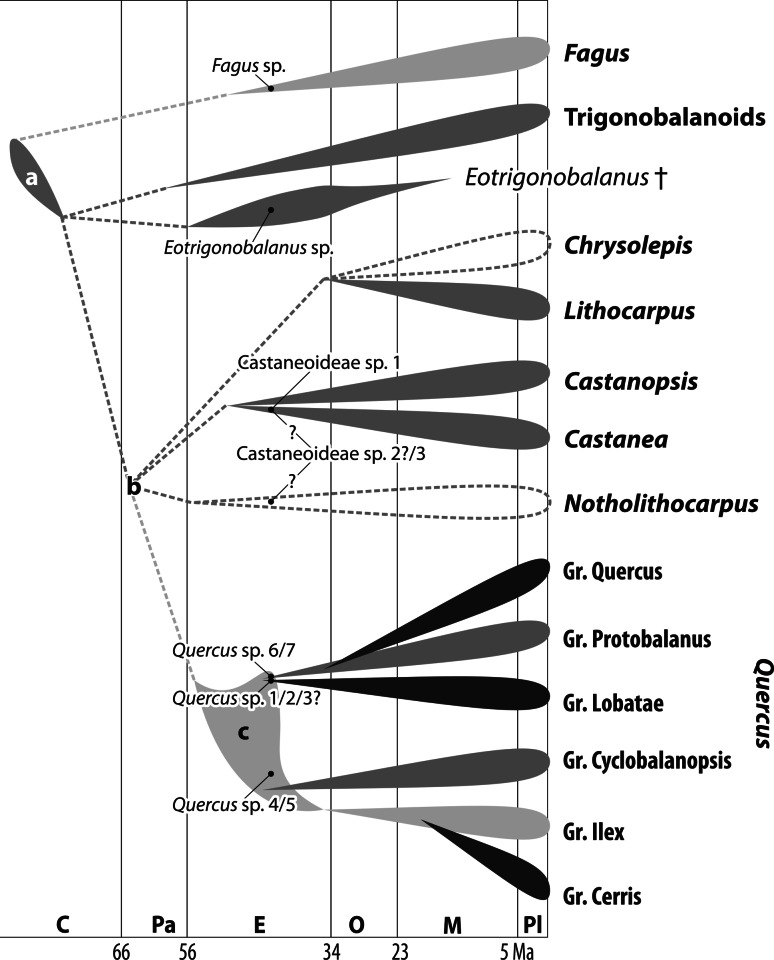
Synopsis of phylogenetic relationships within modern Fagaceae based on several nuclear marker datasets (Oh and Manos 2008; Denk and Grimm 2010; Hipp et al. 2014; Hubert et al. 2014). Ages for major splits are inferred from the fossil record. Lineages for which a good fossil record is available are indicated by grey shading: *light grey* underived (plesiomorphic) rugulate pollen, *dark grey* more derived pollen, rugulate pattern partly masked by sporopollenin, *black* derived pollen with prominent verrucate sculpturing. Lineages without reliable fossil record are indicated by stippled lines. Fossil pollen types from Hareøen are plotted within a temporal frame and based on their systematic affinities. **a** Oldest known fagaceous pollen, showing a distinct castaneoid pollen sculpturing (Takahashi et al. 2008) **b** soft polytomie reflecting ambiguous relationships among the members of crown group Fagaceae (Castaneoideae and Quercoideae); the timing of these splits is not resolved by the fossil record **c** Temporal occurrence of underived, ancestral lineages of *Quercus* (root age from Hubert et al. 2014). Abbreviations: C = Late Cretaceous; Pa = Paleocene; E = Eocene; O = Oligocene; M = Miocene; Pl = Plio-/Pleistocene

Intra-family relationships in Fagaceae are shown in Fig. 16 based on different nuclear DNA sequence data sets (Oh and Manos 2008; Denk and Grimm 2010; Hubert et al. 2014). The temporal framework is based on earliest occurrences of modern lineages (Crepet and Daghlian 1980; Kvaček and Walther 1987; McIntyre 1991; Manchester 1994; Manchester and Dillhoff 2004; Hofmann et al. 2011; Kmenta 2011; Denk et al. 2012; Bouchal 2013); complemented by a recent molecular dating in case of the *Quercus* lineage (Hubert et al. 2014). The plotted pollen record from western Greenland fits perfectly within this independently derived temporal and phylogenetic framework. Pollen representing modern lineages are Castaneoideae sp. 1 and sp. 2, *Fagus* sp., and *Quercus* sp. 1–3. Pollen representing ancestral lineages are *Quercus* sp. 4–7. Castaneoideae sp. 3 may either represent an ancestral, extinct or modern (*Castanea, Castanopsis,* or *Notholithocarpus*) Castaneoideae lineage. Apart from the current lack of an Eocene fossil record of the *Lithocarpus*-*Chrysolepis* lineage, all modern members of *Lithocarpus* are distinctly subtropical-tropical (A, Cfa, Cwa climates, only locally extending into moist Cwb climates, southern foothills of Himalayas and western Yunnan; Menitsky 2005). The same holds true for the putative North American sister taxon of *Lithocarpus*, *Chrysolepis*, a narrow endemic of northern California and Oregon (Csa climate; Thompson et al. 1999). *Notholithocarpus* has a similar geographic range, but occurs up to 2,200 m a.s.l. within the conifer-dominated montane vegetation belt (Csb, Dfb climates). Of the *Castanea*-*Castanopsis* lineage only *Castanea* extends into distinctly temperate Cfb, Csb and Cwb climates in Eurasia (EUFORGEN 2009; Fang et al. 2009). Taken all evidence together, and assuming that all Castaneoideae pollen from Hareøen represent temperate taxa, likely modern analogues appear to be the Eurasian *Castanea* and the western North American *Notholithocarpus.*

Pollen evidence points to the presence of three distinct lineages of *Quercus* in the Eocene of western Greenland. *Quercus* sp. 1–3 most likely represent *Quercus* Group Lobatae. Of these three pollen taxa, *Quercus* sp. 1 and sp. 2 are closely similar to a number of modern species of *Quercus* Group Lobatae. *Quercus* sp. 3 could possibly also represent *Quercus* Group Quercus. Fossil and molecular evidences, however, suggest that *Quercus* Group Quercus may indeed have evolved later from Group Protobalanus like ancestors (Denk and Grimm 2010; Hubert et al. 2014; see Fig. 16). The pollen record and possibly also the leaf record (McIver and Basinger 1999) appear to support this hypothesis. Pollen with affinity to Group Protobalanus is present in the early Cenozoic of western Greenland (*Quercus* sp. 6, 7) and predates the earliest potential record of Group Quercus from the Baltic amber of Poland (ca. 39 Ma; Crepet 1989). Notably, unambiguous representatives of modern members of Group Protobalanus and Quercus are not found co-existing prior to the Eocene–Oligocene boundary in North America (Florissant beds; J. Bouchal et al., unpubl. data), represented by pollen and foliage. The finding of Group Ilex type pollen (*Quercus* sp. 4, 5) may appear surprising in light of the modern ecology and distribution of members of this group and its fossil record (Menitsky 2005; Denk and Grimm 2010; Velitzelos et al. 2014). Denk and Grimm (2009) argued that Group Ilex type pollen is plesiomorphic within the genus, as also evidenced by pollen ontogeny (Rowley and Gabarayeva 2004). The presence of a Group Ilex pollen morphotype in Greenland in a distinctly temperate setting strongly suggests that this pollen belonged to an extinct or ancestral *Quercus* lineage. In addition, two extinct lineages of Fagaceae are present. *Eotrigonobalanus* is an extinct lineage of ambiguous phylogenetic affinity that was widespread during the Palaeogene in the Northern Hemisphere, with a peak in abundance during the late Eocene (Fig. 16; Denk et al. 2012). The other extinct taxon is the leaf genus *Fagopsiphyllum*, a genus that is based on foliage and widespread in northern latitudes during the Palaeogene (Manchester 1999). This taxon was originally described by Heer (1868–1883) as *Quercus groenlandica*. Budantsev and Golovneva (2009) suggested that the taxon should be kept in *Quercus*. In the light of the Fagaceae diversity documented here, it is unlikely that pollen from the *Fagopsiphyllum (Quercus groenlandica)* plant is not represented in our sample: a sample including two major lineages of *Quercus* represented by more or less plesiomorphic pollen types, i.e. Group Ilex pollen morphotype and Group Protobalanus pollen morphotype (*Quercus* sp. 4–7). Morphologically, *Fagopsiphyllum* foliage resembles *Quercus sadleriana* R.Br.ter, a narrow endemic of western North America (northernmost California). Within the modern white oaks, *Q. sadleriana* is genetically unique and a possible evolutionary link between Group Protobalanus and Group Quercus (Hubert et al. 2014). The distribution of *Q. sadleriana* highlights its status as a relict. It is found in nearly the same forest belt as *Notholithocarpus*, being an element of montane coniferous forests from 600–2,200 m a.s.l. (Flora of North America Editorial Committee 1997). Therefore, we speculate that one or both of the *Quercus* Group Protobalanus pollen morphotypes encountered in the present study might actually come from the same plant as the *Fagopsiphyllum (Quercus)* foliage.

### Fagaceae diversity in the Arctic Palaeogene

According to previous studies on Eocene formations of Greenland, Fagaceae did not play an important role in the subarctic vegetation of the Northern Hemisphere (Schloemer-Jäger 1958; Mai 1995; Kvaček 2010). In this study, we report for the first time a diversified fossil record of Fagaceae in the Palaeogene of western Greenland comprising both extinct and modern lineages. Previous records of modern Fagaceae lineages from the Arctic regions have been restricted to middle Eocene (ca. 45 Ma) sediments of Axel Heiberg Island (McIntyre 1991; McIver and Basinger 1999). McIntyre (1991) described pollen of *Fagus*, *Quercus* Group Lobatae/Quercus, and *Castanea*, and McIver and Basinger (1999) figured leaves of *Quercus* Group Lobatae/Quercus and cupules with attached nuts which they referred to as “?*Trigonobalanus*”. The geographic position of the Fossil Forest of Axel Heiberg Island is almost 10° more to the north than Hareøen and has so been during the middle Eocene (Jahren 2007). Furthermore, from Paleocene sediments of Greenland the extinct genera *Eotrigonobalanus* [as *Dryophyllum furcinerve* (Rossmässler) Schmalhausen] (foliage; Mai 1995) and *Fagopsiphyllum* (foliage; Manchester 1999) have been reported; the latter is also known from Spitsbergen (Budantsev and Golovneva 2009). Grímsson et al. (unpublished data) identified unambiguous foliage of *Fagus* from the middle Eocene of Hareøen. The here documented pollen assemblage complements and extends earlier records. The diversity and abundance of fagaceous pollen grains in the Hareøen samples lend credibility to Engler’s original idea of an Arcto-Tertiary Element. During the middle Eocene of Greenland, and possibly in other coeval high-latitude settings (e.g. Kamchatka, Budantsev 1997), forests thrived with a diversity of Fagaceae that is comparable to that in modern temperate, mesophytic forests.

### Origin of the Arcto-Tertiary Element

The rich fossil record of Fagaceae found in the slightly older middle Eocene of Axel Heiberg Island (*Castanea*, *Fagus*, *Quercus*, *Trigonobalanopsis*; Basinger 1991; McIntyre 1991; McIver and Basinger 1999) and the here presented data show that a number of temperate lineages were already diversified by the Eocene. This can also be seen in plant taxa other than Fagaceae. For example, unambiguous records of temperate Sapindaceae (*Acer, Aesculus*), Ulmaceae (*Ulmus*), Juglandaceae (*Juglans*) and others indicate that a substantial proportion of the high-latitude Eocene forest flora was composed of modern lineages in addition to extinct lineages (Budantsev and Golovneva 2009). However, this does not necessarily imply that these temperate groups actually originated in Arctic areas as advocated by Chaney (1959) and Mai (1991). Their concept of an Arcto-Tertiary Geoflora is invalidated by the fact that most, if not all, of these lineages have a fossil record predating the high-latitude (middle Eocene) records. The oldest fossil record of *Fagus* is from the late early Eocene of western North America (Washington, south British Columbia; Manchester and Dillhoff 2004) predating the records from Axel Heiberg and Greenland by at least five million years (Fig. 16). Fossils with strong affinities to modern Castaneoideae (*Castanea*) are also known from the middle Eocene Claiborne Formation (western Tennessee; Crepet and Daghlian 1980). Hubert et al. (2014), using ingroup constraints (fossils that can be assigned to intrageneric lineages), reconstructed a potential root age for the genus *Quercus* of 55 (68–48) Ma that is in good agreement with earliest reliable records of the genus (cupules and acorns, Clarno Formation, Nut Beds, 49 Ma; Manchester 1994, 2011; undifferentiated *Quercus* pollen, Paleocene-Eocene boundary, ca. 55 Ma, St. Pankraz, Austria; Hofmann 2010; Hofmann et al. 2011). Likewise, *Aesculus* dates back to the late Paleocene of North Dakota and Wyoming (ca. 60–55 Ma; Manchester 2001; Zetter et al. 2011); *Ulmus* to the early Eocene of western North America and Northeastern China (ca. 50 Ma; Denk and Dillhoff 2005; Wang et al. 2010); and *Juglans* to the middle Eocene of North America (ca. 44 Ma; Manchester 1987).

## Conclusion

The detection of a hyperdiverse pollen flora in middle Eocene sediments of western Greenland shows that the family Fagaceae played an important role in high-latitude broad-leaved forests of the early Cenozoic. The availability of high-latitude temperate habitats may have promoted radiation, and possibly diversification, of already existing lineages within Fagaceae and other temperate trees. Some of these lineages persist until today (e.g. *Fagus*) and are still dominant forest elements of the northern temperate zone. In addition, important evolutionary processes may have been promoted by these high-latitude forests, which eventually led to the formation of new intrageneric groups (*Quercus*).

Engler’s concept of an Arcto-Tertiary Element dominating the modern forests of the temperate zone is still vital. Taxa that are dominant in these forests were also common elements in the high-latitude forests of the Palaeogene. Chaney’s later concept of an Arcto-Tertiary Geoflora that implies that modern northern temperate forest taxa originated at high latitudes must be entirely rejected (cf. Wolfe 1969, 1972; Spicer et al. 1987). Most Eocene high-latitude elements have so far a mid-latitude record that predates the Arctic one.
